# Remarks upon the Teeth of the Natives of India

**Published:** 1858-03

**Authors:** 


### REMARKS UPON THE TEETH OF THE NATIVES OF INDIA.

[The following very interesting remarks upon the teeth of the inhabitants of India, are from the pen of one who spent a number of years in that country; observing minutely the manners, customs and habits of that people. They use very little animal food, and clense their teeth often, a numbers of times each day, and the result is that they almost uniformly have sound healthy teeth.

Statistics of the conditions and peculiarities of the teeth of different nations are very interesting as they indicate to a very considerable extent the influence of habits, different kinds of food, and manner of living, upon the teeth.

We hope any of our readers who may come in contact with travelers or missionaries from foreign countries, will endeavor to elicit from them facts and statistics, in regard to the teeth of those to whom they have had access.]

Like most travelers in India, I was struck with the glittering whiteness, and admirable preservation of the teeth of the natives. Rarely, if ever, did I remark a mouth disfigured by a disgusting array of black and decayed teeth and stumps, such as so frequently, in our own enlightened land, offends in more ways than one the sense of the dental surgeon. This peculiar ivory-like whiteness, is no doubt, as in the case of the negro, in some degree due to the effect of the contrast with the dusky pair of lips through the parting of which, in smiling or conversation they suddenly gleam. But it is no doubt in a great measure to be accounted for by the fact, that a daily and scrupulous cleaning of the teeth, in common with frequent ablutions of the whole person, is enjoined upon them by their religion, as a duty of the utmost sacredness. I do not remember how often in the course of the day this is required, but I am safe in saying at least twice, namely just before or after the ordinary morning and evening meal. You may be sure they do not accomplish this important operation by means of the cheap, but admirable brushes, with which we are furnished by the ingenious hand of a polished civilization. Indeed, as these are made of the bristles of the hog, in their eyes, the most unclean of all animals, the very touch of an American tooth-brush would be utter pollution to a Hindoo, and by destroying his cast, entail upon him the most horrible consequences. But he finds upon every shrub and tree a very simple and efficacious substitute. Cutting off a tender slip with the knife or little hatchet, he invariably carries as an essential part of his personal outfit, he chews the end until he has thoroughly separated the woody fibres, and then applies it to his teeth with a rigor and a patience that might shame many an individual with greater pretensions to cleanliness. The result of this simple process is the fine polish which so excites the traveler's admiration. An exception must be made to this remark, in behalf of a large class in the lower provinces of India, who are addicted to the habitual chewing of the betel nut, the effect of which is, for the time, to dye the teeth and gums a brilliant scarlet. I have been told that this custom, though attended with this disagreeable discoloration conduces to the healthy condition of both.

Never having made the subject a matter of close investigation, I am not entirely sure how far the teeth of the natives of India, excel those of Europeans or Americans, in the particulars of evenness and regularity, but my strong impression is, that the former would be decidedly the gainers by a comparison.

It would, perhaps, be proper to remark, that the Hindoos are restricted by their religion to a vegetable diet. Whether this has any bearing upon the facts that I have stated, I leave to be decided by the profession.

I may venture to say, however, that my observations on this subject in India, have led me to conclude that sugar, whether in its granulated state or in the form of candy, can not be so injurous to teeth, as popular opinion in this country would make out. The natives of India consume it in every shape, and in quantities that would excite the utmost solicitude in the breast of an American mother, were she to see her boy guilty of a similar habitual indulgence.

In Calcutta and some other large stations in the lower provinces, there are a number of English dentists in successful practice. In the northern and more lately occupied districts, however, I have only met with two of the profession, and I have heard it rumored that their skill was miserably out of proportion with their ordinary charges. The latter were indeed so enormous, that one would find it almost as cheap to come back to this country for a new set of teeth, including the passage both ways, as to depend upon these gentlemen for their over charged services. It is possible that an American of enterprise and skill, would find a professional visit to India a very successful adventure.

I merely add that though in the foregoing remarks, I have only mentioned the Hindoos, all that I have said is equally true of the Mahomedans, who, in this respect, although not required by their religion, have adopted the prevailing customs of the country. L.

